# The need to further understand who gets hospitalized for a COPD exacerbation

**DOI:** 10.1186/2049-6958-7-7

**Published:** 2012-06-19

**Authors:** Diego J Maselli, Antonio Anzueto

**Affiliations:** 1 South Texas Veterans Health Care System, Audie L. Murphy Division; Department of Medicine, Division Pulmonary/Critical Care Medicine, University of Texas Health Science Center at San Antonio, San Antonio, TX, USA

## 

Chronic obstructive pulmonary disease (COPD) has significant morbidity, major impact on health care costs [1] and is the 3rd leading cause of death in the United States [2-4]. The impact becomes greater as the disease progresses, and is mainly due to the development of acute exacerbations and need for hospitalizations [5]. Preventing COPD exacerbations has been a main focus in the treatment of COPD patients. Patients admitted to the hospital for COPD exacerbation are at a higher risk of re-hospitalization and death [6-8]. COPD also increases the hospitalization rate and in-hospital mortality from other co-morbidities [9]. Several therapeutic options including the use of inhaled corticosteroids, long acting bronchodilators, phosphodiesterase inhibitors, pulmonary rehabilitation services, and prolonged antibiotic use have been showed to reduced the incidence of these events [5,10]. Identifying risk factors for hospitalizations have important health care implications, but the knowledge of these is limited.

In this issue of *Multidisciplinary Respiratory Medicine*, García-Sanz et al. [11] reported a prospective observational study conducted in their Emergency Department to determine the frequency of COPD exacerbations, and factors associated with hospitalization. These investigators reported 409 exacerbations in 239 patients (79% male, mean age 75 years); 57% of exacerbations required hospitalization. There were several differences between hospitalized and non-hospitalized patients including history of prior exacerbations, COPD disease severity, use of concomitant medications such as inhaled and systemic corticosteroids, beta-blockers and antibiotics. In the multivariate logistic regression analysis the factors associated with hospitalization were impaired oxygenation (*p<0.001*), increased white blood cells (*p<0.01*) and prescribed antibiotics (*p<0.05*).

This study provides insight into factors associated with hospitalizations for COPD exacerbations in patients that are evaluated in an emergency room. The study was carried out in a single center and therefore the results may be difficult to generalize to other institutions. It is possible that some inherent limitations hampered the ability to find significant differences in several clinical parameters and outcomes. For example, several of the differences in variables between both groups were not significant in the multivariate logistic regression analysis. Some of the results reported in this study are consistent with large cohort publications. For example, patients with prior history of hospitalizations and more severe lung disease were more likely to required hospitalization. These data are similar to the reports by Hurst et al. [5] and Tsoumakidou M. et al. [12], and provide useful clinical data when these patients are evaluated in the emergency department. This study provides practitioners with other useful clinical data that is routinely collected in patients with a severe COPD exacerbation evaluated in the emergency department, such as oxygenation parameters (PaO_2_/FiO_2_ index), and white blood cell counts. COPD severity based on lung function (GOLD stage) was shown to also predicted the risk of hospitalization in COPD for other conditions like pneumonia [4]. Therefore, in this study, patients with a suspicious underlying infectious process, elevated white blood cell count and worsening oxygenation were prescribed antibiotics and were more likely to be hospitalized. It will be interesting if the investigators could provide additional data, such as patients co-morbid conditions, concomitant medications, radiological findings if a chest radiograph was obtained and complications developed during hospitalization. Nevertheless, the data presented by Garcia-Sanz et al. [11] will help to design prospective clinical studies and interventions related to decrease COPD exacerbations related hospitalizations.

Because of the fragile condition of these patients and concomitant morbidity associated with COPD exacerbations, the identification of clinical parameters that indicate patient’s need for hospitalization will have a significant clinical impact. Providing effective clinical care by using routine clinical data represents a new perspective of the overall management of these complex patients. The current study by Garcia- Sanz et al. [11], taken together with existing data from other large observations cohorts, highlights the need to challenge commonly used clinical parameters to make a decision when to hospitalize patients with COPD exacerbations. In our opinion, further clinical studies are needed to help in the management of this critical condition and should take into consideration the results of these investigators.
